# Intrathoracic malignant peripheral nerve sheath tumors: imaging features and implications for management

**DOI:** 10.2478/raon-2013-0047

**Published:** 2013-07-30

**Authors:** Sophia Constance Kamran, Atul Bhanudas Shinagare, Stephanie Anne Holler Howard, Mizuki Nishino, Jason Laurence Hornick, Katherine Margaret Krajewski, Nikhil Himmatsinh Ramaiya

**Affiliations:** 1 Department of Imaging, Dana-Farber Cancer Institute, 450 Brookline Ave, Boston, MA 02115, USA; 2 Department of Radiology, Brigham and Women’s Hospital, 75 Francis Street, Boston, MA 02115, USA; 3 Department of Pathology, Brigham and Women’s Hospital, 75 Francis Street, Boston, MA 02115, USA; 4 Harvard Medical School, Boston MA 02115, USA

**Keywords:** malignant peripheral nerve sheath tumors, chest, neurofibromatosis, imaging, malignant triton tumor

## Abstract

**Background:**

The aim of the study was to analyze the clinical and imaging characteristics of primary intrathoracic malignant peripheral nerve sheath tumors (MPNSTs).

**Patients and methods.:**

In this institutional review board (IRB)-approved retrospective study, clinical and imaging features of 15 patients (eight men; mean age 50 years [range 18–83)] with pathologically proven malignant peripheral nerve sheath tumors seen from January 1999 to December 2011 were analyzed. Imaging features (CT in 15, MRI in 5 and PET/CT in 4) of primary tumors were evaluated by three radiologists and correlated with clinical management.

**Results:**

Of the 15 tumors, six were located in the mediastinum (two each in anterior, middle and posterior mediastinum), four in chest wall, two were paraspinal, and three in the lung. Four patients had neurofibromatosis-1 (NF1); four tumors had heterologous rhabdomyoblastic differentiation (malignant triton tumor). Masses typically were elongated along the direction of nerves, with mean size of 11 cm. The masses were hypo- or isodense to muscles on CT, isointense on T1-weighted images, hyperintense on T2-weighted images and intensely fluorodeoxyglucose (FDG) avid (mean standardized uptake value [SUV]_max_ of 10.5 [range 4.4–23.6]). Necrosis and calcification was seen in four tumors each. Finding of invasion of adjacent structures on imaging led to change in management in seven patients; patients with invasion received chemoradiation.

**Conclusions:**

Intrathoracic MPNSTs appear as large elongated masses involving mediastinum, lung or chest wall. Radiological identification of invasion of adjacent structures is crucial and alters therapy, with patients with invasion receiving neoadjuvant or adjuvant chemoradiation.

## Introduction

Malignant peripheral nerve sheath tumors (MPNSTs) are rare, accounting for 5–10% of all soft tissue sarcomas.^1^ MPNSTs usually demonstrate nerve sheath differentiation, and although they grossly appear similar to benign peripheral nerve sheath tumors such as schwannomas and neurofibromas, MPNSTs commonly contain hyperchromatic spindle cells with nuclear atypia, mitotic activity, and areas of tumor necrosis on histology.^1^,^2^ Rapid enlargement, pain, or associated neurological symptoms favor MPNST over a benign nerve sheath tumor.^1^ MPNSTs are most often sporadic, although 20–30% occur in association with neurofibromatosis-1 (NF1).^1^ Patients typically present around age 40–50^1^, although patients with NF1 often present earlier (mean age 30 years).^3^ MPNSTs most often occur in the extremities^1^, followed by trunk and head and neck, however MPNSTs can arise anywhere in the body. Intrathoracic MPNST are particularly rare.^4^ Pathologically, MPNSTs typically have a fascicular growth pattern and are composed of spindle cells with variable nuclear atypia and mitotic activity. MPNSTs with heterologous rhabdomyoblastic (*i.e*. skeletal muscle) differentiation are called malignant triton tumors (MTTs). MPNSTs have an aggressive clinical course with 5- and 10-year survival rates of 34–60% and 22–45% respectively.^1^,^5^ MTT is associated with an even worse clinical outcome, with a 5-year survival rate of 11–15%.^6^ Location also plays a role in outcome, with tumors in the trunk associated with poorer prognosis, likely attributable to difficulty in achieving negative surgical margins and consequent local or regional recurrence.^1^,^5^ While studying the impact of rhabdomyoblastic differentiation (MTT), presence of NF1 and location on the outcome of 84 consecutive patients with MPNST^7^, we noticed that the imaging literature on intrathoracic MPNST is extremely limited, consisting mostly of case reports with little information on imaging findings.^4^,^8^–^14^ Therefore, the purpose of this study was to assess the clinical and imaging characteristics of primary intrathoracic MPNST.

## Patients and methods

### Patients

In this institutional review board-approved study, the electronic pathology patient database was reviewed to identify patients with pathologically proven primary intrathoracic MPNSTs (mediastinum, lung, and chest wall tumors) evaluated at our institution from January 1999 to December 2011. First, we identified a total of 110 patients with pathologically proven MPNSTs. Among them, 26 patients were excluded because they were referred from outside institution and the pathology of the primary tumor was not confirmed by review at our institution. Among the remaining 84 patients, 21 patients had intrathoracic MPNSTs. Of these 21 patients, 6 were excluded because imaging of the primary tumor was not available. Therefore, the final study population consisted of 15 patients with pathologically proven intrathoracic MPNST who had imaging of primary tumors available for review. Mediastinal tumor localization was determined by an adaptation of the Felson methodology. For large tumors, we used the epicenter of the tumor to define its location. Clinical features including clinical presentation and treatment were correlated with imaging findings.

### Image Analysis

All imaging analysis was performed, in consensus, by three radiologists (N.R., S.H. and A.S.) with 13, 10 and 7 years of experience. Images were reviewed on the picture archiving and communication system (PACS) using commercially available workstations (Centricity, General Electric, Barrington, IL).

A systematic review of available imaging studies was performed and location, size (single longest dimension was selected by manually placing caliper on the axial or coronal images), and imaging features (CT in 15, MRI in 5 and PET/CT in 4) of the primary tumors were recorded. CT attenuation of the tumor was compared to muscles by placing a circular region of interest (ROI) in the most representative portion of the tumor. Image intensity on T1 and T2-weighted images was visually compared to muscles. Degree of enhancement was evaluated in comparison with skeletal muscle and graded as mild if less enhancement than skeletal muscle, moderate if similar degree of enhancement as muscle (considered similar for the purpose of this study if the ROI values were within 10% the muscle), and intense if more enhancement than muscle using ROI measurements, similar to previous reports.^15^ Homogeneous or heterogeneous appearance of the tumors on CT and MRI, presence of hemorrhage (defined as areas that are hyperdense on unenhanced CT images or T1-hyperintense areas on T1-weighted MR images), necrosis (defined as hypodense and/or T2-hyperintense, non-enhancing area within the tumor), and calcifications were also recorded. Degree of fluorodeoxyglucose (FDG) uptake was measured on PET/CT images by manually placing a circular ROI over the most FDG avid portion of the lesion on a commercially available workstation (Hermes, Hermes Medical Solutions Inc, Greenville, NC, USA). Standardized uptake value (SUV)_max_ was used to represent the FDG uptake of the tumor. Invasion of adjacent structures, seen as extension of tumor invading and distorting the adjacent tissues, was noted and its impact on management was studied.

Differentiation between malignant and benign nerve sheath tumors is challenging, especially in NF1 patients. Signs of malignancy in this context on imaging include high attenuation and necrosis/hemorrhage on CT, heterogeneity on T1 weighted images in MR, and rapidly increasing size.^16^,^17^

## Results

### Patients

Among 15 eligible patients, there were eight men and seven women (Table 1). The average age at diagnosis was 49 years (range: 18–83 years). Eleven patients had conventional MPNSTs, and 4 patients had MTT (27%). The average age of the MPNST patients was 50 (range 18–83) while the average age of the MTT patients was 40 (range 27–67). Four patients (27%) had NF1 (2 patients with MPNST, 2 patients with MTT).

**FIGURE 1.**
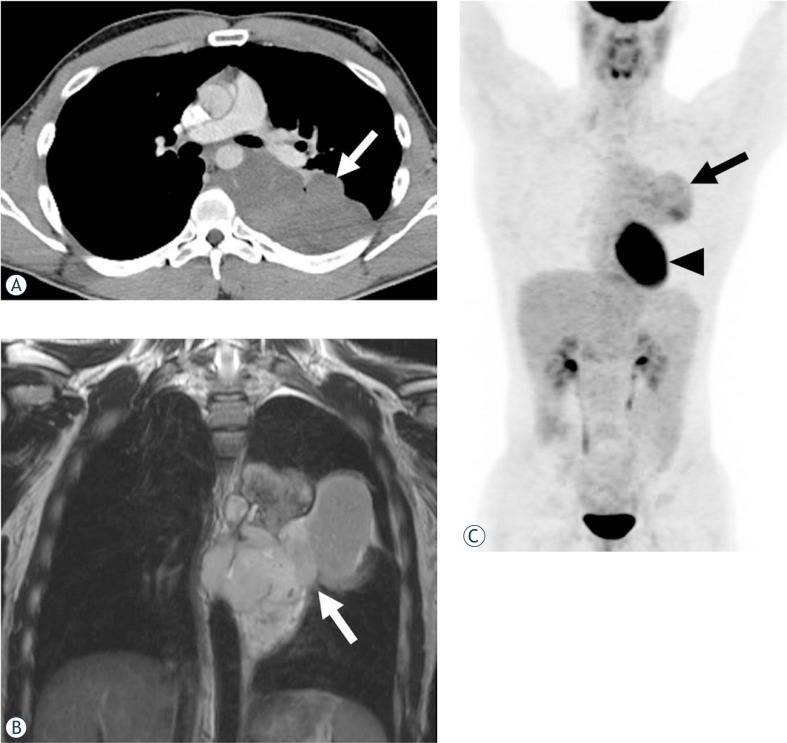
A 31-year-old man with mediastinal malignant peripheral nerve sheath tumor. **A.** Axial contrast-enhanced CT (CECT) shows a low attenuating mildly enhancing posterior mediastinal mass (arrow) displacing the aorta, azygous vein and esophagus. The mass encompasses more than 50% circumference of the descending thoracic aorta. **B.** Coronal T2-weighted MR image shows a heterogeneous lobulated hyperintense mass (arrow). **C.** Coronal maximum intensity projection (MIP) PET image shows a moderately fluorodeoxyglucose (FDG) avid mediastinal mass (arrow, standardized uptake value [SUV]_max_ 5.2). Physiologic activity is noted in the heart (arrowhead).

**FIGURE 2.**
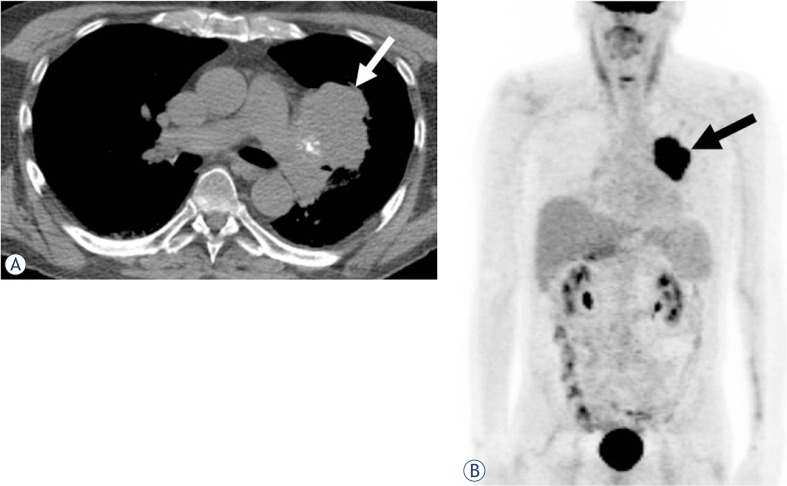
A 67-year-old man with MPNST involving the left lung. **A.** Unenhanced CT image of the chest at the level of main pulmonary arteries shows a lobulated mass abutting the left hilum with peripheral foci of calcification (arrow). **B.** Coronal maximum intensity projection (MIP) image from PET/CT shows an intensely fluorodeoxyglucose (FDG) avid mass (arrow, standardized uptake value [SUV]_max_ 23.6). Patient underwent left pneumonectomy on which intrapulmonary location of the mass was confirmed. Rhabdomyoblastic differentiation (malignant triton tumor) was noted on pathology.

**FIGURE 3.**
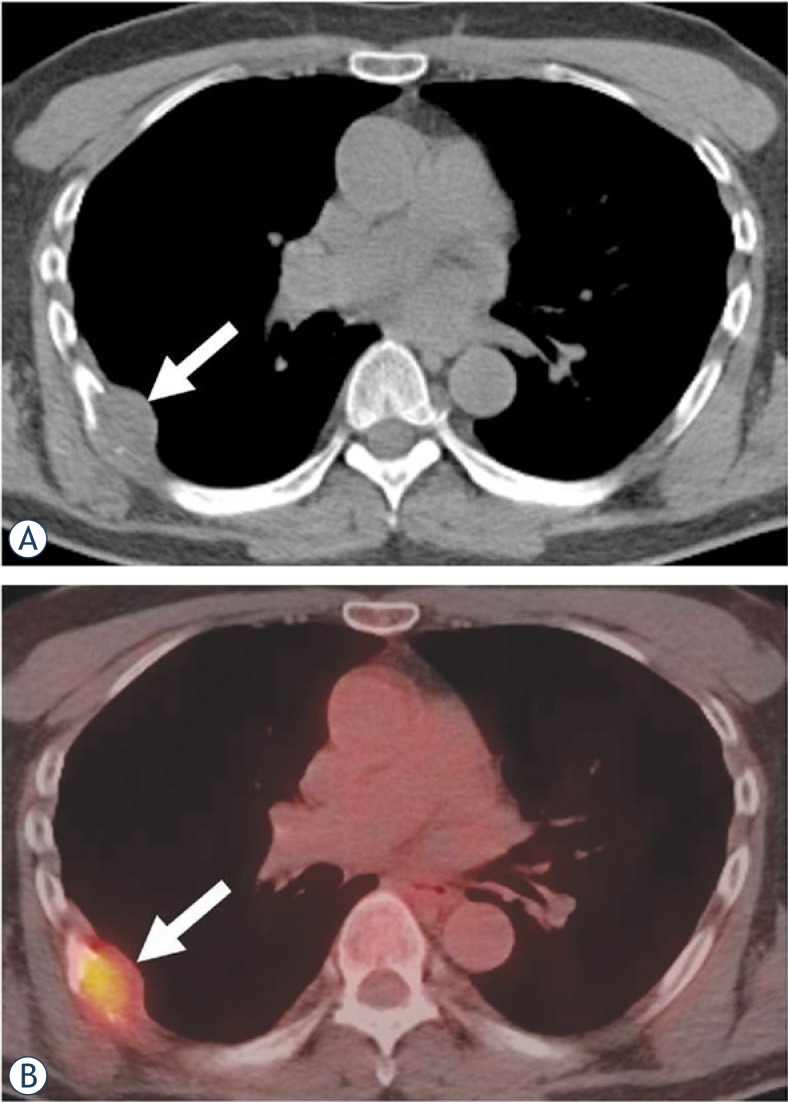
A 60-year-old man with left chest wall MPNST. **A.** Axial unenhanced CT image shows a low attenuation right chest wall mass abutting a rib (arrow) causing rib destruction and adjacent pleural thickening. **B.** Axial fused PET/CT image shows presence of moderate fluorodeoxyglucose (FDG) uptake (arrow, standardized uptake value [SUV]_max_ 4.4).

Six tumors (40%) were located in the mediastinum (two anterior, two middle and two in posterior mediastinum) (Figure 1), four (27%) were in the chest wall (Figure 2), three (20%) involved the lung, and two were paraspinal (13%) (Figure 3).

Of the six mediastinal tumors, all were conventional MPNSTs and 2 were associated with NF1 (33%). Of the four chest wall tumors, 2 were MTT, both of which were associated with NF1 (50%), while the other two were conventional sporadic MNPST. Of the three lung tumors, 1 was a MTT (33%); none of these patients had NF1. Of the two paraspinal tumors, 1 was a MTT (50%) and none of the patients had NF1.

Nine patients presented with a chief complaint of pain, three with back pain, three with pleuritic chest pain, two with painful chest wall lumps, and one with right arm pain. Six patients presented with dyspnea or respiratory distress. One patient presented with right arm tingling/swelling. Table 2 summarizes the clinical presentation of the patients with intrathoracic MPNST. Only one patient was asymptomatic, with a large subxiphoid mass detected on surveillance imaging; the patient had received chemotherapy and radiation for Hodgkin’s lymphoma 14 years prior to developing an MPNST. Clinical information regarding presentation was unavailable for one patient.

### Imaging features of primary tumors

All patients had a single tumor representing MPNST. Average tumor size was 11 cm (range 3–32 cm) (Table 3). Patients with MTT tended to have larger tumors at diagnosis (mean size 15 cm) than those with conventional MPNSTs (mean size 9 cm). However, this difference could not be statistically assessed due to a small sample size.

On CT, eight tumors (53%) had lower attenuation than muscle and seven (47%) were isoattenuating. On MRI, all five masses were isointense to muscles on T1-weighted images and hyperintense on T2-weighted images. Ten masses demonstrated mild enhancement, four had moderate enhancement, and one patient only had unenhanced CT. Eight (53%) masses were heterogeneous and seven (47%) were homogeneous.

Calcifications were seen in four lesions (27%) and four (27%) had features suggestive of necrosis. Three patients had punctate calcifications and one had chunky, nodular calcifications; calcifications were peripheral in two patients, central in one patient, and diffusely scattered in one. Of the four masses with appearance suggestive of necrosis on imaging, two had necrosis on pathology and one had cystic degeneration. No necrosis or cystic degeneration was noted on pathology in the fourth patient. Hemorrhage was seen in two masses (13%).

The four masses that underwent PET/CT were intensely FDG––avid with mean SUV_max_ of 10.5 (range 4.4–23.6). One of these had MTT, and we noticed that this tumor had higher SUV_max_ (23.6) than those without MTT (mean 6.1, range 4.4–8.9). Again, this difference could not be statistically analyzed given the small number of patients with this rare entity.

Neuroforaminal extension was seen in one posterior mediastinum patient and one paraspinal patient. Two patients had evidence of rib erosion on imaging, while two patients had evidence of rib displacement. Of the six mediastinal cases, four had invasion or encasement of adjacent structures on imaging such as trachea, mediastinal great vessels and esophagus, and one had intraspinal extension. Of the chest wall cases, one had invasion of adjacent tissues (4 cm) and another large mass had extensive invasion and displacement of multiple adjacent structures. All three of the lung cases had invasion of nearby structures including chest wall, diaphragm and mediastinum. Of the paraspinal cases, one patient presented with lung metastases, and the other patient had invasion into paraspinal musculature and extrapleural fat.

### Management

In general, surgery was the treatment of choice whenever possible. Large masses which were initially deemed unresectable received neoadjuvant chemotherapy or chemoradiation, following which they were surgically resected if there was response and if they were considered resectable. Patients with nonradical resection received adjuvant chemotherapy.

Two posterior mediastinal masses and one middle mediastinal mass were treated with neoadjuvant chemoradiation or chemotherapy followed by surgery. The patient with the 14cm posterior mediastinal mass received concurrent chemotherapy (etoposide/ifosfamide) and radiation followed by surgical resection, as the tumor was initially deemed unresectable. One patient with middle mediastinal mass and another with an anterior mediastinal mass underwent chemoradiation alone as they were deemed unresectable. The patient with the anterior mediastinal mass received chemotherapy (etoposide/cisplatin) followed by radiation, the patient with the middle mediastinal mass received concurrent chemotherapy (adriamycin/ifosfamide) and radiation. Another patient with a 4 cm subxiphoid mass was resected without neoadjuvant therapy.

Of the four patients with chest wall masses, the 4 cm invasive mass and another large mass (17 cm) underwent surgery and chemoradiation, and one small mass (3 cm) was treated with surgical resection. The patient with the 4 cm invasive mass initially underwent non-radical resection, followed by radiation therapy, re-resection, and then chemotherapy with vincristine/actinomycin/cyclophosphamide/mesna; exact details of chemotherapy were not available for the 17cm chest wall mass as patient received chemotherapy at an outside institution. The one patient with the 32 cm mass and extensive invasion of adjacent structures died prior to any treatment.

Of the three lung tumors, one case with mediastinal invasion was treated with neoadjuvant chemotherapy followed by surgery, one case with a chest wall and diaphragmatic invasion was treated with chemotherapy, and a hilar mass with invasion of the pulmonary artery was treated with total pneumonectomy.

Of the two paraspinal cases, one patient was treated with chemoradiation (further details not available), while the other was treated with chemotherapy alone, as the patient presented initially with widespread metastatic disease.

## Discussion

Thoracic MPNSTs may involve mediastinum, lung or chest wall, with those in the mediastinum or chest wall manifesting as large elongated masses. They are often heterogeneous on CT and MRI, and intensely FDG-avid on PET. To our knowledge, this is the largest study of imaging findings of intrathoracic MPNST.

MPNSTs are rare, aggressive soft tissue sarcomas occurring anywhere in the body. They often occur in association with NF1, with an incidence ranging from 20–30%.^1^,^3^,^5^ Four patients in our study had NF1 (27%); this number is consistent with the reported incidence. MPNSTs most commonly occur in the extremities; intrathoracic MPNST are rarely reported, mostly in the form of isolated case reports. If MPNSTs arise in the nerves of the deep tissue, as occurs frequently with thoracic tumors, early diagnosis is clinically more difficult.^4^ Pain was the most common complaint and presenting symptom in others (7/15 patients). In a recent series of 175 patients with MPNSTs conducted at Mayo Clinic, the median tumor size was 6.0 cm^1^; our average tumor size was 11 cm.

MTT, defined by the presence of rhabdomyoblasts in an MPNST on pathology, account for < 10% of MPNSTs.^6^ MTTs behave more aggressively than conventional MPNSTs. Out of the 15 patients in our study, 4 had the MTT subtype (27%). These patients had a larger tumor size at diagnosis, measuring 15 cm compared to 9 cm for conventional MPNSTs.

MPNSTs typically are ovoid, occurring along a nerve^18^, and presenting as a non-specific soft tissue mass. MPNSTs have low attenuation on CT.^9^ On MR, MPNSTs are isointense on T1 and hyperintense on T2.^8^ Our findings for intrathoracic MPNST are similar, as 8/15 patients had hypoattenuating lesions on CT, and of the five patients with MR, all had isointense lesions on T1 and hyperintense lesions on T2. MPNSTs present as FDG-avid masses and can therefore be evaluated using PET/CT.^19^

Differentiation between malignant and benign nerve sheath tumors is challenging, especially in NF1 patients. Signs of MPNST on imaging include high attenuation and necrosis/hemorrhage on CT, heterogeneity on T1 weighted images in MR, and rapidly increasing size.^16^,^17^ A “target sign”, or a central hypodense region on T2-weighted images, was previously implicated as an indicator for MPNST, but this has not been corroborated by other groups.^2^,^20^,^21^ Our data also did not confirm this finding, as a target sign was only seen in one patient. MPNST should be considered in the differential diagnosis for large thoracic masses found on imaging, especially if they are located along major nerves. MPNST should be especially high on the differential diagnosis if a thoracic mass is found in a patient with NF1.

Treatment of MPNST relies primarily on surgical resection.^4^ Neoadjuvant chemoradiation is often used for initially unresectable tumors with an intention to downstage the mass pre-resection. Radiation therapy can be given in addition to surgical resection for improved local control.^5^ Chemotherapy is typically reserved for aggressive cases, and also when there is tumor rupture, positive margins, or metastases, although improvement in survival has not been demonstrated.^1^ In an analysis of MPNST patients who received either surgery alone or multimodality treatment, the rate of recurrence was not significantly affected^7^, however, given the aggressive nature of MPNSTs, multimodality treatment is typically recommended. To the best of our knowledge, there are no studies comparing the efficacy of sequential versus concurrent chemoradiation in the treatment of MPNSTs.

Imaging assists with management of these patients, as tumors visualized to have invasion or compression of nearby structures often undergo additional therapy prior to surgical resection. PET/CT may play an important role in MPNST management, as increased FDG uptake is associated with malignant transformation, and PET/CT has 72% specificity for diagnosis of malignancy.^19^ In addition, whole-body MRI adequately assesses tumor burden in patients with NF1, and can allow visualization of tumors not observed on physical examination. This technique allows close surveillance of neurofibromas in NF1 patients and may allow for earlier detection of malignant transformation.^21^,^23^

An important limitation to this study was small sample size. However, intrathoracic MPNST are rare, and there are no prior reports on imaging features of series of these tumors. Due to the retrospective nature of the study with patients undergoing imaging evaluation as clinically indicated, we do not have MRI or PET/CTs for all 15 patients. As these are rare tumors, there is no fixed protocol for imaging evaluation of these masses.

In summary, intrathoracic MPNST may involve the mediastinum, lung and chest wall; mediastinal and chest wall tumors are usually large elongated masses along the course of nerves. They are usually hypoattenuating on CT, hyperintense on T2-weighted images, are often heterogeneous, and are intensely FDG-avid masses. Comparison with previously published data on MPNSTs suggests that intrathoracic MPNST are similar in appearance to MPNSTs in other locations, but these tumors may present larger in size. For MPNSTs, imaging is often nonspecific, and although we as radiologists can raise the possibility, an accurate diagnosis always needs histologic confirmation. MPNST should be considered in the differential diagnosis when a large, elongated intrathoracic mass is found. The role of the radiologist lies in identification of invasion of adjacent structures as patients with invasion receive neoadjuvant or adjuvant chemoradiation.

## Figures and Tables

**TABLE 1.** Clinical features of patients with malignant peripheral nerve sheath tumor (MPNST) of chest

**Sr No**	**Age**	**Sex**	**NF1**	**Location**	**Size (cm)**	**Histology (MPNST/MTT)**	**Management**
1	38	F	Yes	Middle mediastinum	16	MPNST	Chemoradiation
2	31	M	No	Posterior mediastinum	14	MPNST	Neoadjuvant chemoradiation, followed by surgery
3	48	M	No	Anterior mediastinum	13.6	MPNST	Chemoradiation
4	18	F	Yes	Middle mediastinum	8	MPNST	Neoadjuvant chemoradiation, followed by surgery
5	65	M	No	Posterior mediastinum	5.5	MPNST	Neoadjuvant chemotherapy and surgery
6	33	F	No	Anterior mediastinumsubxiphoid	4	MPNST	Surgery
7	83	F	No	Chest wall	17	MPNST	Surgery, followed by chemoradiation
8	60	M	No	Chest wall	3	MPNST	Surgery
9	27	M	Yes	Chest wall	4	MTT	Surgery, followed by chemoradiation
10	44	F	Yes	Chest wall	32	MTT	None
11	51	F	No	Left lung	4.5	MPNST	Neoadjuvant chemotherapy and surgery
12	63	M	No	Left lung	unknown	MPNST	Chemotherapy
13	67	M	No	Left lung	12.8	MTT	Surgery
14	58	M	No	Paraspinal	2.5	MPNST	Chemoradiation
15	61	F	No	Paraspinal	10	MTT	Chemotherapy

**TABLE 2.** Initial presentation of patients with malignant peripheral nerve sheath tumor (MPNST) of the chest

**Sr No**	**Location**	**Size (cm)**	**Histology (MPNST/MTT)**	**Presentation**
1	Middle mediastinum	16	MPNST	Right arm pain with radiation to axilla
2	Posterior mediastinum	14	MPNST	Back pain
3	Anterior mediastinum	13.6	MPNST	Dyspnea, found to have mass with partial obstruction of right mainstem bronchus
4	Middle mediastinum	8	MPNST	Dyspnea/pleuritic chest pain and pleural effusion
5	Posterior mediastinum	5.5	MPNST	Back pain
6	Anterior mediastinum-subxiphoid	4	MPNST	Asymptomatic, found on routine screening
7	Chest wall	17	MPNST	Right arm tingling/swelling
8	Chest wall	3	MPNST	Painful chest wall lump
9	Chest wall	4	MTT	Painful chest wall lump
10	Chest wall	32	MTT	Dyspnea/pleuritic chest pain and pleural effusion
11	Left lung	4.5	MPNST	PNA, tx’d, with continuing decline and respiratory distress
12	Left lung	unknown	MPNST	Dyspnea/pleuritic chest pain and pleural effusion
13	Left lung	12.8	MTT	Unknown
14	Paraspinal	2.5	MPNST	Back pain
15	Paraspinal	10	MTT	Lung mets and dyspnea on exertion

MTT = malignant triton tumors; Sr No = serial number

**TABLE 3.** Imaging features of patients with malignant peripheral nerve sheath tumor (MPNST) of the chest

**Sr No**	**Location**	**CT density**	**MR T1**	**MR T2**	**Enhancement**	**Homogeneity**	**Calcification**	**Necrosis**	**Hemorrhage**	**PET SUVmax**
1	Middle mediastinum	Hypo	-	-	Mild	Homogenous	Yes	-	-	-
2	Posterior mediastinum	Hypo	Iso	Hyper	Moderate	Heterogenous	-	-	Yes	5.2
3	Anterior mediastinum	Hypo	-	-	Mild	Homogenous	-	-	-	-
4	Middle mediastinum	Iso	Iso	Hyper	Mild	Heterogenous	-	-	-	-
5	Posterior mediastinum	Iso	Iso	Hyper	Moderate	Heterogenous	-	-	-	-
6	Anterior mediastinum-subxiphoid	Hypo	-	-	Mild	Heterogenous	-	-	-	-
7	Chest wall	Iso	-	-	Mild	Heterogenous	Yes	Yes	Yes	-
8	Chest wall	Iso	-	-	Mild	Homogenous	-	-	-	4.4
9	Chest wall	Iso	Iso	Hyper	Moderate	Homogenous	-	-	-	-
10	Chest wall	Hypo	-	-	Mild	Heterogenous	-	Yes	-	-
11	Left lung	Hypo	-	-	Mild	Heterogenous	Yes	Yes	-	-
12	Left lung	Hypo	-	-	Moderate	Homogenous	-	Yes	-	8.9
13	Left lung	Iso	-	-	-	Homogenous	Yes	-	-	23.6
14	Paraspinal	Iso	Iso	Hyper	Mild	Homogenous	-	-	-	-
15	Paraspinal	Hypo		-	Mild	Heterogenous	-	-	-	-

Sr No = serial number; SUV_max_ = standardized uptake value
